# Clust&See3.0 : clustering, module exploration and annotation

**DOI:** 10.12688/f1000research.152711.1

**Published:** 2024-09-02

**Authors:** Fabrice Lopez, Lionel Spinelli, Christine Brun

**Affiliations:** 1 TAGC (UMR1090), Aix-Marseille Université, INSERM, Turing Centre for Living Systems, Marseille, 13009, France; 2 INSERM-CNRS, CIML, Turing Centre for Living Systems,, Aix-Marseille Univ, Marseille, France; 3 CNRS, Marseille, 13009, France

**Keywords:** interaction networks, graph partitioning, clustering, visualization, cluster annotations, functional modules, statistical enrichment.

## Abstract

**Background:**

Cytoscape is an open-source software to visualize and analyze networks. However, large networks, such as protein interaction networks, are still difficult to analyze as a whole.

**Methods:**

Here, we propose Clust&See3.0, a novel version of a Cytoscape app that has been developed to identify, visualize and manipulate network clusters and modules. It is now enriched with functionalities allowing custom annotations of nodes and computation of their statistical enrichments.

**Results:**

As the wealth of multi-omics data is growing, such functionalities are highly valuable for a better understanding of biological module composition, as illustrated by the presented use case.

**Conclusions:**

In summary, the originality of Clust&See3.0 lies in providing users with a complete tool for network clusters analyses: from cluster identification, visualization, node and cluster annotations to annotation statistical analyses.

## Introduction

Several years ago, we proposed Clust&See, a Cytoscape ^1^ plug-in that aims to facilitate network clustering and analysis for biologists by providing several original functionalities within a single framework. ^2^ Mainly, the tool allows decomposing a network into disjoint or overlapping clusters using several in-house algorithms, ^3^^–^^5^ visualizing those clusters as metanodes linked by several types of edges/relationships, and manipulating the clusters for further detailed visualization, exploration, analyses and comparisons. We have now developed Clust&See3.0 to fit Cytoscape3 new API and added functionalities allowing the user to annotate the nodes and the clusters with orthogonal data as well as to compute statistical enrichments for those annotations. We here provide examples of Clust&See3.0 usage on a protein-protein interaction network, where annotation enrichments are used *(i)* to functionally annotate the clusters with the Gene Ontology terms describing node functions in order to globally investigate the network and proceed with protein function prediction, and *(ii)* to discover features associated to clusters by the integration of data on nodes.

## Methods

### Implementation

Briefly, the classic use of Clust&See ^2^ breaks down into the following phases:

### Decompose a network

As in its first version, Clust&See proposes to partition an imported network using three algorithms: FT (Fusion-Transfer), an ascending hierarchical method fusing two clusters iteratively if the fusion results in a modularity gain ^3^; TFIT (iterated Transfer-Fusion), ^4^ a multi-level algorithm in which a vertex transfer procedure is performed to the best adjacent cluster while modularity increases, to finally compute a quotient graph. Both algorithms generate strict network partitions where clusters have no node in common. The third one, OCG (Overlapping Cluster Generator) ^5^ is an ascending hierarchical method fusing two clusters at each step while modularity increases, starting from an overlapping class system. This leads to overlapping clusters where some nodes belong to several clusters.

### Cluster visualization and exploration

For each cluster in a partition, Clust&See provides detailed information about the cluster: a visualization of the cluster's sub-network, and its main topological features (number of nodes, edges, density, etc.)

**Figure 1.**
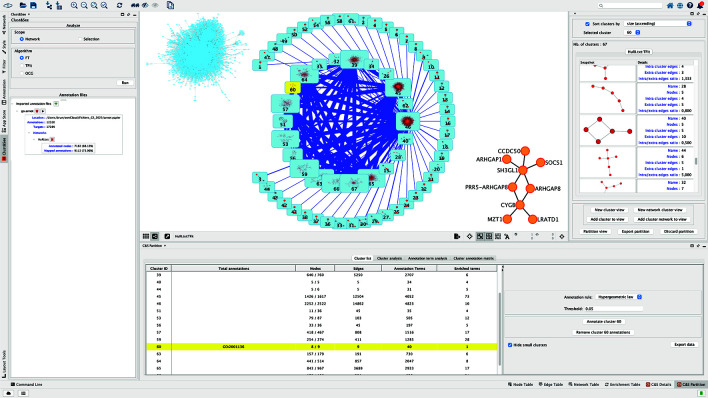
Clust&See3.0 interface. The central panel shows (1) the starting network in the top left corner, (2) the quotient graph after TFit partitioning where modules are represented by blue nodes with the icon of the corresponding sub-network inside. The size of the node reflects the number of nodes contained by the module. The blue links correspond to the edges linking the modules, their width reflecting the number of involved interactions. (3) In the right low corner, module 60 is shown as an expanded network. As in the former version of Clust&See, the right panel shows the details of the subnetworks forming the modules with their characteristics. The lower panel (former Data panel) contains the results of the different Cluster and Annotation analyses.

Each cluster can be viewed and explored independently, either in a compact mode as a metanode, or in an extended mode as the sub-network constituting the cluster ( Figure 1). The decomposition of the complete network can be viewed as a set of metanodes linked by edges, the thickness of which is proportional to the number of links between pairs of nodes of the linked metanodes. When clusters are overlapping, another type of link is added between the metanodes to represent the nodes shared between clusters/metanodes. The thickness of this link is then proportional to the number of shared nodes. From this map, the metanodes can be individually switched to the extended mode to visualize the corresponding sub-network, and back. Finally, the user can build a custom map by iteratively adding sub-networks and/or metanodes to obtain a global view of the partition ( Figure 1, central panel).

The novel version of Clust&See3.0 now allows the user to annotate and analyze the nodes and the clusters as follows:

### Importing node annotations

In order to annotate the clusters, Clust&See3.0 allows importing one or more annotation lists, composed of associations between a network node and one or more terms of any nature (see the Use case).

The annotation file must be a text file and must contain at least two columns: one contains the node identifier (one identifier per line), the second one, the list of terms associated with this identifier, separated by a comma, a semicolon or a tab. The columns can also be separated by a comma, semicolon or tab, as long as the column separator is not the same as the term separator. The annotation import dialog box lets the user select the 2 columns of interest and eliminate any header lines.

After import, Clust&See3.0 provides the number and percentage of annotated nodes in the network. Then the annotation process can be performed.

### Annotation rules, statistical enrichment

Two types of enrichment can be computed for each annotation term, using the whole graph as background:

• A one-sided hypergeometric hypothesis test, the null hypothesis of which corresponds to the proportional distribution of the annotation terms between the nodes inside and the nodes outside the cluster. When the p-value of the hypergeometric test is sufficiently low to reject the null hypothesis, this indicates that the nodes of the cluster carry the term more frequently than expected by chance, thus pointing toward a potential enrichment. It should be noted that since this test is applied to all clusters, the Benjamini-Hochberg procedure ^6^ is used to correct for the multiple testing effect on the p-values. The default value is set to p-values < 5.10 ^-2^.
• A majority rule, corresponding to a minimum percentage (to be chosen by the user) of nodes annotated to the term among the annotated nodes of the cluster. The default value is set to 50%.

### Cluster and annotation analyses

**Figure 2.**
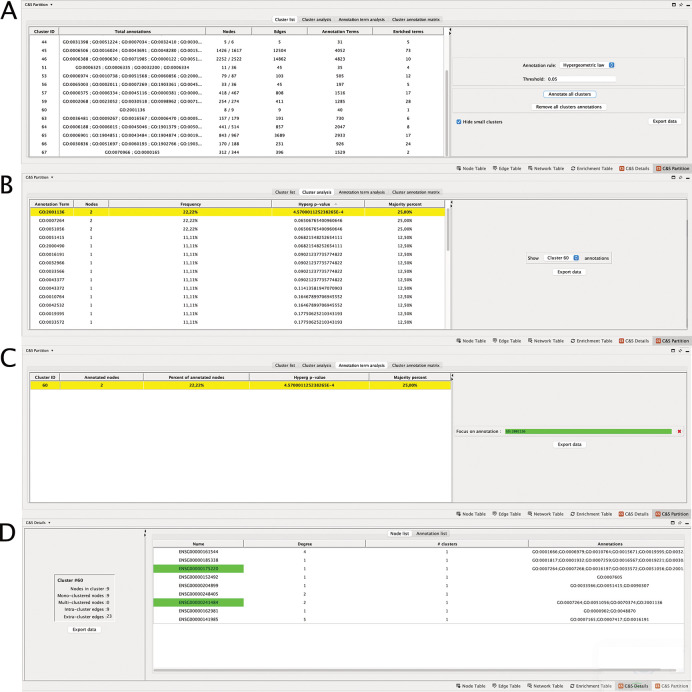
Details of the different tabs of the Data panel. (A) “Cluster List” shows the annotation of all clusters; (B) “Cluster Analysis”, all the annotations of cluster 60; (C) “Annotation term analysis”, all the clusters annotated to GO:2001136; (D) “Node list”, the detail of the annotations of each nodes of the cluster. The node names highlighted in green are those annotated to the term of interest, here GO:2001136.

For each annotation list, the user can perform a statistical analysis of the clusters and the annotation terms, with different goals. At first, a global analysis of the annotations of the partition can be performed. When choosing the “Cluster list” tab, Clust&See3.0 provides for each cluster, *(i)* the number of nodes in the cluster that have received at least one annotation term, *(ii)* the number of annotation terms appearing at least once in the cluster, *(iii)* the number of terms that are statistically enriched in the cluster with a hypergeometric test, or that annotate a majority of cluster nodes ( *i.e.* with a “majority rule”). For both tests, thresholds are set by the user ( Figure 1). Finally, *(iv)* the terms that are enriched in clusters are shown on demand ( Figure 2A). This first type of analysis allows getting a global view of the annotation distribution and to quickly identify clusters that are enriched for annotation terms of interest.

The second approach concerns the details of statistical analyses by cluster by choosing the “Cluster analysis” tab. Here, the user can select a particular cluster for a detailed study of the annotations of its nodes. Clust&See3.0 then lists the terms annotating the cluster proteins and provides *(i)* the number of nodes annotated to the term, *(ii)* the percentage of nodes annotated to the selected term among the total number of cluster nodes, *(iii)* the p-value of the term according to the hypergeometric test, *(iv)* the percentage of nodes annotated to the selected term among the total number of annotated cluster nodes ( Figure 2B).

The third approach concerns the details of the statistical analyses by annotation term. When choosing the “Annotation term analysis” tab, the user can select a particular annotation term and Clust&See3.0 reports for each cluster that contains proteins annotated to this term, the same features than previously ( Figure 2C). This type of analysis allows having a detailed view on the distribution of a particular annotation term among all the clusters composing the network.

The tables displaying the results for the clusters are dynamic and can be used to identify the clusters selected in the map and, in the panel detailing the characteristics of the clusters. The reverse is also true: a selected cluster in the map or in the detailed panel is selected in the annotation statistical results tables.

### Operation

The minimum system requirements for use of the Clust&See3.0 Cytoscape app include:

**Hardware:**

Memory: 8 GB

Monitor: 1600×900 (HD+) resolution

**Software:**

Java 11 and above

Cytoscape 3.8.0 and above

## Use Case

### Annotation mode for network and cluster exploration, and for function prediction

We will illustrate how to use Clust&See3.0 by partitioning and annotating the human reference interactome network (HuRI) ^7^ with Gene Ontology ^8^ (network and annotation files are available at https://doi.org/10.5281/zenodo.12570870). We first partitioned the largest connected component of the HuRI network that contains 8149 nodes and 52016 edges, with the TFit algorithm. ^4^ Sixty-seven modules were obtained among which 20 contain more than 4 nodes ( Figure 1). After loading the annotation file that contains the list of IDs of the Biological Process Gene Ontology (BP GO) associated to all human genes/proteins, the number of annotated nodes in the network is indicated: 88% of the proteins of HuRI have functional annotations in the BP GO.

• “Cluster list” tab

In the “C&S Partition” table, under the “Cluster list” tab, all clusters are shown ( Figure 2A). As we empirically consider that the smallest clusters are not suitable for computing statistics on annotations, Clust&See3.0 proposes to hide them for clarity’ sake with a check box “Hide small clusters”.

The number of enriched terms per cluster according to the chosen statistic (“Hypergeometric law” or “Majority rule”) is indicated, and their GO IDs are available in the “Total annotations” column ( Figure 2A) (choose “Annotate cluster X/Remove cluster X annotations” or “Annotate all clusters/Remove all cluster annotations”). The list of annotation terms also appears by right clicking on a cluster of interest on the Partition view, under “Clust&See>Annotate cluster”. At this stage, custom annotations can be added manually by the user to tag a cluster of interest. This annotation will also appear in the list of annotation terms in the “Total annotations” column of the “Cluster list” table. This may help having a global quantitative view of the cluster annotations and further analyses.

The individual investigation of a particular cluster starts also under this tab. For instance, Cluster 60 contains 8/9 nodes annotated (number given in the “Nodes” column), and solely 1 term is statistically enriched among the 40 terms (number given in the “Annotation terms” column) that annotate the proteins of the cluster, when the hypergeometric law with a corrected p-value threshold set at 5.10 ^−2^ is chosen.

• “Cluster analysis” tab

The term GO:2001136 is enriched among the annotations of the proteins of cluster 60 with a corrected p-value of 4,57.10 ^-4^, available in “Hyperg p-value” column under the “Cluster analysis” tab ( Figure 2B). The most relevant term ( *i.e.* with the lowest p-value) is easily found by using the ranking column containing the corrected p-values for the terms annotating all the proteins of cluster 60. The term GO:2001136 that corresponds to “negative regulation of endocytic recycling”, is annotating 25% of the annotated proteins of the cluster (number in the “Majority percent” column). Two other terms are also annotating 25% of the cluster’s proteins, but with highest p-values *i.e.* less significant ones. These are GO:0007264 “small GTPase-mediated signal transduction” and GO:0051056 “regulation of small GTPase mediated signal transduction”. Then, switching to the “C&S Details” Table ( Figure 2D), under the “Node” tab, that shows the detailed annotations of the nodes of the cluster, we see that the three terms are associated to the same two genes encoding the proteins involved in these functions: ENSG00000175220 (ARHGAP1) and ENSG00000241484 (ARHGAP8). By expanding the cluster 60 on the network panel ( Figure 1, central panel), it can be seen that these two proteins do not interact directly but with the product of ENSG00000141985 (SH3GL1), a protein regulating endocytosis by recruiting proteins to the membrane.

• “Annotation term analysis” tab

Then, wondering whether other clusters are also annotated to “negative regulation of endocytic recycling”, we switched to the “Annotation term analysis” tab to focus on a particular annotation and find all clusters enriched for this term. By entering the ID GO:2001136 in the dedicated frame, we found that none of the clusters but cluster 60 is annotated to this term, either using the hypergeometric law or the majority rule computations ( Figure 2C).

The user can therefore choose to annotate this cluster with this enriched term and, if appropriate, to transfer the annotation/function to any not yet annotated node of the cluster. Notably, the nodes contributing to cluster’s annotations are detailed in the “C&S Details” table ( Figure 2D).

Export of the annotations and computations as text files are available at each step, as well as a matrix of the whole results under the “Cluster annotation matrix” tab, for further analysis.

## Conclusion/Discussion

Clust&See3.0 is a Cytoscape app that allows *(i)* clustering the nodes of any network, *(ii)* annotating the clusters with any annotation terms, and *(iii)* computing their enrichment significance. This versatility of Clust&See3.0 is constituting its advantage compared to other existing Cytoscape enrichment plug-ins. Not only Clust&See3.0 allows as in its previous version loading any partition of the network, even not generated by the app, as long as the graph is completely covered by clusters, but it also allows using any type of data as node annotations, such as user’s experimental or curated data. In contrast, most of the existing apps are mainly centered on Gene Ontology terms or other types of classical annotations (i.e. ^8^^,^^9^). Second, the results of Clust&See3.0 permit to get a global view of the distribution of the annotations between the whole set of clusters, as well as their statistical value. For all these reasons, we think Clust&See3.0 will be a valuable tool for the community.

## Ethics and consent

Ethics and consent not required.

## Data Availability

Zenodo: Clust&See3.0: clustering, module exploration and annotation. https://doi.org/10.5281/zenodo.12570870. The project contains the following underlying data:
• go_annot.txt: The protein annotation file extracted from Gene Ontology Biological Process database. ^10^
• HuRI_CC.txt: The network file containing the largest connect component of the human reference interactome network. ^7^ Data are available under the terms of the Creative Commons Attribution 4.0 International license (CC-BY 4.0).

## Software availability

• Software available from: https://apps.cytoscape.org/apps/clustnsee3
• Source code available from: https://github.com/fafa13/ClustnSee-3
• Archived source code at time of publication: https://doi.org/10.5281/zenodo.13220735. ^11^
• License: GNU General Public Licence, V3
