# Supplementary material for: Clust&See3.0 : clustering, module exploration and annotation
Source: F1000Res. 2024 Nov 14;13:994. Originally published 2024 Sep 2. [Version 2] doi: 10.12688/f1000research.152711.2 (PMC11528184; doi:10.12688/f1000research.152711.2)
Supplement: Peer review history

# Reviewer report: Reviewer response for version 2

**DOI:** 10.5256/f1000research.174224.r340656

**Published:** 2024-12-13

**Authors:** Lun Hu

**Affiliations:** 1 Xinjiang Technical Institute of Physics and Chemistry, Chinese Academy of Sciences, Urumqi, China

All of my concerns have been addressed in this revision.

Are the conclusions about the tool and its performance adequately supported by the findings presented in the article?

No

Is the rationale for developing the new software tool clearly explained?

Yes

Is the description of the software tool technically sound?

No

Are sufficient details of the code, methods and analysis (if applicable) provided to allow replication of the software development and its use by others?

No

Is sufficient information provided to allow interpretation of the expected output datasets and any results generated using the tool?

No

Reviewer Expertise:

Complex network analysis

I confirm that I have read this submission and believe that I have an appropriate level of expertise to confirm that it is of an acceptable scientific standard.

# Reviewer report: Reviewer response for version 2

**DOI:** 10.5256/f1000research.174224.r340655

**Published:** 2024-11-19

**Authors:** Anooja Ali

**Affiliations:** 1 REVA University, Bengaluru, Karnataka, India

No further comments

Are the conclusions about the tool and its performance adequately supported by the findings presented in the article?

Yes

Is the rationale for developing the new software tool clearly explained?

Yes

Is the description of the software tool technically sound?

Yes

Are sufficient details of the code, methods and analysis (if applicable) provided to allow replication of the software development and its use by others?

Yes

Is sufficient information provided to allow interpretation of the expected output datasets and any results generated using the tool?

Yes

Reviewer Expertise:

Bioinformatics, Deep learning , Computer Vision, Data mining, Computational Biology

I confirm that I have read this submission and believe that I have an appropriate level of expertise to confirm that it is of an acceptable scientific standard.

# Reviewer report: Reviewer response for version 1

**DOI:** 10.5256/f1000research.167505.r320337

**Published:** 2024-10-31

**Authors:** Lun Hu

**Affiliations:** 1 Xinjiang Technical Institute of Physics and Chemistry, Chinese Academy of Sciences, Urumqi, China

In this paper, the authors propose Clust&See3.0, a novel version of a Cytoscape app that has been developed to identify, visualize and manipulate network clusters and modules. Here are some comments that may be helpful.

 1. The introduction section needs to be further strengthened. Since the proposed Clust&See is a Cytoscape for network clustering and analysis of biological data, the authors should provide relevant background and current needs. This will help to better illustrate the significance and applicability of the Cytoscape.

 2. In the methods section, for the use of Clust&See3, the authors should provide a general process. Particularly, the differences between Clust&See3 and Clust&See should be emphasized.

 3. The novel version of cluster&see3.0 allows users to annotate and analyze nodes and clusters, which is indeed very favorable. However, in the paper, the authors should focus on the advantages and implications of doing so.

 4. In the conclusion section, the authors should briefly discuss the limitations and future improvements of Clust&See3.0.

Are the conclusions about the tool and its performance adequately supported by the findings presented in the article?

No

Is the rationale for developing the new software tool clearly explained?

Yes

Is the description of the software tool technically sound?

No

Are sufficient details of the code, methods and analysis (if applicable) provided to allow replication of the software development and its use by others?

No

Is sufficient information provided to allow interpretation of the expected output datasets and any results generated using the tool?

No

Reviewer Expertise:

Complex network analysis

I confirm that I have read this submission and believe that I have an appropriate level of expertise to confirm that it is of an acceptable scientific standard, however I have significant reservations, as outlined above.

# Response

**Published:** 2024-11-25

**Authors:** Christine Brun

**Affiliations:** Aix-Marseille University- INSERM- CNRS, France

Dear Referee,

 We thank you for your positive review of our manuscript and acknowledge your remarks on our work.

 Please find below our point-by-point response.

  

1. *The introduction section needs to be further strengthened. Since the proposed Clust&See is a Cytoscape for network clustering and analysis of biological data, the authors should provide relevant background and current needs. This will help to better illustrate the significance and applicability of the Cytoscape.*

The current revised version of the introduction is answering this remark. The introduction has been modified to underline the needs.

 “Although Cytoscape Apps allowing the identification of clusters such as ClusterMaker ^6^, cluster annotations and computation of enrichment statistics such as BinGO ^7^, or combinations of those as proposed by Functional Enrichment Collection ( https://apps.cytoscape.org/apps/functionalenrichmentcollection) do exist, those proposing in a single tool the identification of clusters and their annotation with custom annotation files are seldom. We therefore felt there was room for a tool such as Clust&See3.0, that performs all in a single workflow.”

  

1. *In the methods section, for the use of Clust&See3, the authors should provide a general process. Particularly, the differences between Clust&See3 and Clust&See should be emphasized.*

The Method section is following the general process: 1- Decompose a network, 2- Cluster Visualization and exploration, 3- Importing nodes annotations, 4- Annotation rules, statistical enrichments, 5- Cluster and annotation analyses.

 While the two first steps were already possible with the first version of Clust&See, the step 3 to 5 are specific to Cluts&See3.0. Clust&See3.0 provides the handling of the annotations.

 In fact, Clust&See is included within Clust&See3.0 but has been re-coded to fulfill the Cytoscape3 format.

*3. The novel version of cluster&see3.0 allows users to annotate and analyze nodes and clusters, which is indeed very favorable. However, in the paper, the authors should focus on the advantages and implications of doing so.*

 As explained in the manuscript, the advantage of annotating clusters with Clust&See3.0 is the possibility of using custom annotations, allowing the analysis to exactly fit the will and the question of the user. Then,

 “For each annotation list, the user can perform a statistical analysis of the clusters and the annotation terms, with different goals. At first, a global analysis of the annotations of the partition can be performed. “…”This first type of analysis allows getting a global view of the annotation distribution and to quickly identify clusters that are enriched for annotation terms of interest.”

*4. In the conclusion section, the authors should briefly discuss the limitations and future improvements of Clust&See3.0.*

 The limitations of Clust&See3.0 could reside in the performances of the clustering algorithms. However, all our partitioning/clustering algorithms have been assessed previously by us and others. For instance, the performances of the 3 algorithms (i.e. FT, TFit, OCG) have been recognized to outperform 4 other algorithms on different biological datasets by measuring 5 performance indices (1). The comparison of OCG performances with other algorithms has also been performed by us, in the original publication (2) and by others (3).

 1.      Sharma P, Ahmed HA, Roy S, Bhattacharyya DK. Unsupervised methods for finding protein complexes from PPI networks. Netw Model Anal Health Inform Bioinforma. 2 juin 2015;4(1):8.

 2.      Becker E, Robisson B, Chapple CE, Guénoche A, Brun C. Multifunctional proteins revealed by overlapping clustering in protein interaction network. Bioinformatics. 1 janv 2012;28(1):84‑90.

 3.      Ding Z, Zhang X, Sun D, Luo B. Overlapping Community Detection based on Network Decomposition. Sci Rep. 12 avr 2016;6(1):24115.

  

- ***Is the rationale for developing the new software tool clearly explained?***

*Yes*

- ***Is the description of the software tool technically sound?***

*No*

- ***Are sufficient details of the code, methods and analysis (if applicable) provided to allow replication of the software development and its use by others?***

*No*

 As stated in the manuscript, the code is available on github and zenodo : https://github.com/fafa13/ClustnSee-3 , https://doi.org/10.5281/zenodo.13220735

- ***Is sufficient information provided to allow interpretation of the expected output datasets and any results generated using the tool?***

*No*

- ***Are the conclusions about the tool and its performance adequately supported by the findings presented in the article?***
- *** ***
*No*

On the behalf of all authors,

 Christine Brun

# Reviewer report: Reviewer response for version 1

**DOI:** 10.5256/f1000research.167505.r329845

**Published:** 2024-10-23

**Authors:** Ju Xiang

**Affiliations:** 1 Central South University, Changsha, Hunan, China

The authors reported Clust&See3.0, a new version of Cytoscape app “Clust&See” for the identification, visualization and manipulation of network clusters. It provided functionalities allowing custom annotations and computation of statistical enrichments. It may be a useful tool of network cluster analyses.   

 --It would be better to provide introduction for other tools of the same type in section introduction.

 --It is suggested to provide a table for comparing the different versions of Clust&See and other tools of the same type.  --It is helpful for users to choose and use tools if the complexity of time and space for the algorithms in the tool can be provided.

 --Furthermore, it would be even better if comparisons with other tools could be provided.

Are the conclusions about the tool and its performance adequately supported by the findings presented in the article?

Yes

Is the rationale for developing the new software tool clearly explained?

Yes

Is the description of the software tool technically sound?

Yes

Are sufficient details of the code, methods and analysis (if applicable) provided to allow replication of the software development and its use by others?

Partly

Is sufficient information provided to allow interpretation of the expected output datasets and any results generated using the tool?

Yes

Reviewer Expertise:

Bioinformatics, Data mining, ComputationalBiology

I confirm that I have read this submission and believe that I have an appropriate level of expertise to confirm that it is of an acceptable scientific standard, however I have significant reservations, as outlined above.

# Response

**Published:** 2024-10-29

**Authors:** Christine Brun

**Affiliations:** Aix-Marseille University- INSERM- CNRS, France

Dear Referee,

 We thank you for your positive review on our manuscript and acknowledge your remarks on our work.

 Please find below our point-by-point response.

*The authors reported Clust&See3.0, a new version of Cytoscape app “Clust&See” for the identification, visualization and manipulation of network clusters. It provided functionalities allowing custom annotations and computation of statistical enrichments. It may be a useful tool of network cluster analyses.*

 We thank the reviewer to recognize the utility of our work.

*--It would be better to provide introduction for other tools of the same type in section introduction.*

 Cytoscape Apps proposing in a single tool the identification of clusters AND their annotation with custom annotation files are seldom. We add this information in the introduction section in a new paragraph.

 “Although Cytoscape Apps allowing the identification of clusters such as ClusterMaker ^6^, cluster annotations and computation of enrichment statistics such as BinGO ^7^, or combinations of those as proposed by Functional Enrichment Collection ( https://apps.cytoscape.org/apps/functionalenrichmentcollection) do exist, those proposing in a single tool the identification of clusters and their annotation with custom annotation files are seldom. We therefore felt there was room for a tool such as Clust&See3.0, that performs all in a single workflow.”

*--It is suggested to provide a table for comparing the different versions of Clust&See and other tools of the same type.*

 The previous Clust&See version is completely included in the new one. The differences between the two App versions lie in the statistical enrichment functionalities newly implemented in Clust&See3.

 Regarding other tools of the same type, the comparison is difficult for several reasons. As explain now in the text, other tools propose either to compute clusters, or to annotate them (one by one). Then collections such as Functional Enrichment Collection proposes to install several of these individual tools at once, but the user must perform the transfer of the results of the first tool to the second, etc., whereas Clust&See3.0 provided everything at once. Finally, CyCommunityDetection detect communities and annotate all clusters but not with custom annotations. In addition, it runs on a remote sever whereas Clust&See3.0 is locally installed, thus avoiding issues due to server accessibility or network connection.

*--It is helpful for users to choose and use tools if the complexity of time and space for the algorithms in the tool can be provided.*

 OCG runs in O(n ^3^) with default parameters. The two others run faster. In an independent evaluation (9), we can read “OCG is an elite algorithm of high time efficiency…”

*--Furthermore, it would be even better if comparisons with other tools could be provided.*

 As explained previously, a formal comparison is difficult, mainly because the purpose of the tools being different, the functionalities are different. However, all our partitioning/clustering algorithms have been assessed previously by us and others. For instance, the performances of the 3 algorithms (i.e. FT, TFit, OCG) have been recognized to outperform 4 other algorithms on different biological datasets by measuring 5 performance indices (1). The comparison of OCG performances with other algorithms has also been performed by us, in the original publication (2) and by others (3).

 1.      Sharma P, Ahmed HA, Roy S, Bhattacharyya DK. Unsupervised methods for finding protein complexes from PPI networks. Netw Model Anal Health Inform Bioinforma. 2 juin 2015;4(1):8.

 2.      Becker E, Robisson B, Chapple CE, Guénoche A, Brun C. Multifunctional proteins revealed by overlapping clustering in protein interaction network. Bioinformatics. 1 janv 2012;28(1):84‑90.

 3.      Ding Z, Zhang X, Sun D, Luo B. Overlapping Community Detection based on Network Decomposition. Sci Rep. 12 avr 2016;6(1):24115.

 On behalf of the authors,

 Christine Brun

# Reviewer report: Reviewer response for version 1

**DOI:** 10.5256/f1000research.167505.r320335

**Published:** 2024-09-10

**Authors:** Anooja Ali

**Affiliations:** 1 REVA University, Bengaluru, Karnataka, India

The authors  propose Clust&See3.0, an improved version of Cytoscape, for visualizing and manipulating the bioinformatic network clusters.

 It has additional functionalities , when compared to Cytoscape like custom annotations of nodes. 

 It provided more statistical analysis for cluster evaluation.

 How does the user interface of Clust&See3.0 compare to previous versions in terms of usability and accessibility for biologists unfamiliar with network analysis?

 What are the key characteristics of clusters identified in large protein-protein interaction networks using Clust&See3.0? How do these characteristics correlate with biological functions?

 How does the annotation and enrichment analysis provided by Clust&See3.0 contribute to the accuracy of protein function predictions in complex biological networks?

 I hope the user feedback on Clust&See3.0's functionalities inform future updates and enhancements to the software.

 The citations refer to the aligners for creating a huge protein network based on functional similarities among proteins. The next citation uses cytoscape tool with KEGG pathway analysis and GO annotation tool for generating biclusters. It deals with the generation of gene ontology clusters for each category of MF, BP and CC from biclusters.

Are the conclusions about the tool and its performance adequately supported by the findings presented in the article?

Yes

Is the rationale for developing the new software tool clearly explained?

Yes

Is the description of the software tool technically sound?

Yes

Are sufficient details of the code, methods and analysis (if applicable) provided to allow replication of the software development and its use by others?

Yes

Is sufficient information provided to allow interpretation of the expected output datasets and any results generated using the tool?

Yes

Reviewer Expertise:

Bioinformatics, Deep learning , Computer Vision, Data mining, Computational Biology

I confirm that I have read this submission and believe that I have an appropriate level of expertise to confirm that it is of an acceptable scientific standard.

## References

[1] : Detection of Gene Ontology Clusters Using Biclustering Algorithms. *SN Computer Science* .2023;4(3) : 10.1007/s42979-022-01624-w 10.1007/s42979-022-01624-w

[2] : An Optimization on Bicluster Algorithm for Gene Expression Data. *IEEE explore* .2023; 10.1109/GCAT59970.2023.10353373 10.1109/GCAT59970.2023.10353373

[3] : Pareto Optimization Technique for Protein Motif Detection in Genomic Data Set. *Springer link* .2023; 10.1007/978-981-99-5166-6_65 10.1007/978-981-99-5166-6_65

# Response

**Published:** 2024-10-29

**Authors:** Christine Brun

**Affiliations:** Aix-Marseille University- INSERM- CNRS, France

Dear Referee,

 We thank you for your positive review on our manuscript and acknowledge your remarks on our work.

 Please find below our point-by-point response.

*The authors  propose Clust&See3.0, an improved version of Cytoscape, for visualizing and manipulating the bioinformatic network clusters.*

*It has additional functionalities , when compared to Cytoscape like custom annotations of nodes.*

*It provided more statistical analysis for cluster evaluation.*

*- How does the user interface of Clust&See3.0 compare to previous versions in terms of usability and accessibility for biologists unfamiliar with network analysis?*

**Author response: **The previous version of Clust&See had been developed as an app for Cytoscape2.8, which had a completely different architecture and is almost not used anymore. The new Clust&See3.0 has been completely re-developed to comply with Cytoscape3 architecture. Clust&See3.0 therefore benefits in term of usability and accessibility for biologists from the Cytoscape3 features. Note that Cytoscape3 is far more intuitive than Cytoscape2.

 In addition, to increase the usability of Clust&See3.0 user interface compared to the previous version, we have significantly improved the interconnection of graphical elements and their responsiveness to user actions, so that the user's experience can be enhanced.

*- What are the key characteristics of clusters identified in large protein-protein interaction networks using Clust&See3.0? How do these characteristics correlate with biological functions?*

**Author response: **Both versions of Clust&See propose the same 3 algorithms to identify clusters: FT, TFit and OCG. Most of the clusters found by these algorithms display a biological homogeneity, i.e. they contain proteins involved in the same pathway, the same biological process. This is illustrated by our own network biology analyses in different contexts (1–7) as well as by other authors: the algorithms provided by Clust&See have been used in more than 40 analyses (49 citations in Google Scholar since its first publication (8)). 

*- How does the annotation and enrichment analysis provided by Clust&See3.0 contribute to the accuracy of protein function predictions in complex biological networks?*

**Author response: **The advantage of Clust&See3 is twofold. First, any annotation file can be loaded in Clust&See3, allowing the user to highlight clusters of interest at a glance. Second, according to the goal of the analysis, the user can choose between (i) the accuracy of the annotation using the hypergeometric law (e.g. to highlight clusters containing nodes annotated with rare terms) and (ii) the homogeneity of the annotation with the majority rule (e.g. to highlight clusters containing nodes annotated with the same terms).

 1.      Kim DK, Weller B, Lin CW, Sheykhkarimli D, Knapp JJ, Dugied G, et al. A proteome-scale map of the SARS-CoV-2-human contactome. Nat Biotechnol. janv 2023;41(1):140‑9.

 2.      Katsogiannou M, Andrieu C, Baylot V, Baudot A, Dusetti NJ, Gayet O, et al. The functional landscape of Hsp27 reveals new cellular processes such as DNA repair and alternative splicing and proposes novel anticancer targets. Mol Cell Proteomics. déc 2014;13(12):3585‑601.

 3.      Zanzoni A, Spinelli L, Braham S, Brun C. Perturbed human sub-networks by Fusobacterium nucleatum candidate virulence proteins. Microbiome. 10 août 2017;5(1):89.

 4.      Zanzoni A, Spinelli L, Ribeiro DM, Tartaglia GG, Brun C. Post-transcriptional regulatory patterns revealed by protein-RNA interactions. Sci Rep. 13 mars 2019;9(1):4302.

 5.      Zanzoni A, Brun C. Integration of quantitative proteomics data and interaction networks: Identification of dysregulated cellular functions during cancer progression. Methods. 2016;93:103‑9.

 6.      Ribeiro DM, Zanzoni A, Cipriano A, Delli Ponti R, Spinelli L, Ballarino M, et al. Protein complex scaffolding predicted as a prevalent function of long non-coding RNAs. Nucleic Acids Res. 25 janv 2018;46(2):917‑28.

 7.      Ribeiro DM, Prod’homme A, Teixeira A, Zanzoni A, Brun C. The role of 3’UTR-protein complexes in the regulation of protein multifunctionality and subcellular localization. Nucleic Acids Res. 9 juill 2020;48(12):6491‑502.

 8.      Spinelli L, Gambette P, Chapple CE, Robisson B, Baudot A, Garreta H, et al. Clust&See: a Cytoscape plugin for the identification, visualization and manipulation of network clusters. BioSystems. août 2013;113(2):91‑5.

 On behalf of the authors,

 Christine Brun
